# ICE-CBF-COR Signaling Cascade and Its Regulation in Plants Responding to Cold Stress

**DOI:** 10.3390/ijms23031549

**Published:** 2022-01-28

**Authors:** Delight Hwarari, Yuanlin Guan, Baseer Ahmad, Ali Movahedi, Tian Min, Zhaodong Hao, Ye Lu, Jinhui Chen, Liming Yang

**Affiliations:** 1 College of Biology and the Environment, Nanjing Forestry University, Nanjing 210037, China; tondehwarr@njfu.edu.cn (D.H.); yuanlinguan@njfu.edu.cn (Y.G.); dr.baseerahmadkhan@gmail.com (B.A.); ali_movahedi@njfu.edu.cn (A.M.); tmin@njfu.edu.cn (T.M.); 2 College of Forestry, Nanjing Forestry University, Nanjing 210037, China; haozd@njfu.edu.cn (Z.H.); luye@njfu.edu.cn (Y.L.)

**Keywords:** cold stress, *Inducer of CBF Expression*, *C-repeat Binding Factor*, cold response genes, transcription factors, plant

## Abstract

Cold stress limits plant geographical distribution and influences plant growth, development, and yields. Plants as sessile organisms have evolved complex biochemical and physiological mechanisms to adapt to cold stress. These mechanisms are regulated by a series of transcription factors and proteins for efficient cold stress acclimation. It has been established that the *ICE-CBF-COR* signaling pathway in plants regulates how plants acclimatize to cold stress. Cold stress is perceived by receptor proteins, triggering signal transduction, and *Inducer of CBF Expression* (*ICE*) genes are activated and regulated, consequently upregulating the transcription and expression of the *C-repeat Binding Factor* (*CBF*) genes. The *CBF* protein binds to the *C-repeat/Dehydration Responsive Element* (*CRT/DRE*), a homeopathic element of the *Cold Regulated* genes (*COR* gene) promoter, activating their transcription. Transcriptional regulations and post-translational modifications regulate and modify these entities at different response levels by altering their expression or activities in the signaling cascade. These activities then lead to efficient cold stress tolerance. This paper contains a concise summary of the *ICE-CBF-COR* pathway elucidating on the cross interconnections with other repressors, inhibitors, and activators to induce cold stress acclimation in plants.

## 1. Introduction

Cold stress diminishes plant growth, development, yield, and the geographical distribution of crops, liable for ~40% harvest reduction of crops in temperate regions [1]. It is estimated that extreme cold stress causes between 51–82% of annual crop yield losses globally [2]. Cold stress has been categorized into chilling stress (0–15 °C) and freezing stress (<0 °C) depending on plant effects [3] Cold receptors localized in the plant plasma membrane perceive cold stress stimulus. Instantly, a progression of cell reactions and sub-atomic system adjustments are triggered, remodeling plant physiological, biochemical, and molecular mechanisms for cold stress tolerance through the regulatory actions of numerous transcription factors [4,5,6]. The three main cold-responsive genes in plants are *Inducer of CBF Expression* (*ICE*), *C-repeat Binding Factors* (*CBF*s), and the *Cold-Regulated genes* (*COR*s) [7]. These three forenamed key players, *ICE*, *CBF*, and *COR* genes, model an imperative signaling pathway, the *ICE-CBF-COR* cascade, a cold response pathway that alleviates cold stress in plants [8,9,10,11,12]. Usually, plant cold stress tolerance is characterized by a decrease in plant water losses, reduced plant growth, decreased photoperiod, and other physiological changes [13]. To date, several plant species genomes have been characterized and the *ICE-CBF-COR* cascade has been identified in rice [14], wheat [10,15], and tea [16]. 

The *ICE* acts upstream to induce and regulate the expression of the *C-repeat Binding Factor* (*CBF*) [8,9,10,11]. Consequently, the *CBF*s otherwise known as the *DREB1* genes, regulate cold stress by binding to the *cold and dehydration regulatory elements* (*CRT/DRE*) in the promoter regions of *COR* genes to induce their expression; for instance, *COR15A* [17,18] and *RD29A* [19] in Arabidopsis. Thus, *CBFs* trigger and regulate the expression of *COR* genes under cold stress. Amongst these three aforementioned genes, perhaps *CBF* genes are the most vital cold response factors in plants, other researchers have also published diverse roles and responses in different plant species played by the C-repeat Binding Factor/dehydration-responsive element-binding 1 (*CBF/DREB1*) genes [10,12]. It is established that *CBFs* (*CBF1, CBF2*, and *CBF3*) have different roles under cold stress due to their several modifications in their individual protein sequences, although they have similar sequence structures and binding properties [1,10,20]. Two homologs of *ICE* genes (*ICE1* and *ICE2*) have been characterized in many plant species and their cold tolerance roles were deduced [21]. The activity of the *ICE1* is mainly regulated at the protein level by post-transcriptional and/or post-translational modifications (PTMs). Recent research has shown the importance of PTMs in regulating the *ICE*-*CBF* cascade pathway during cold stress [22,23,24]. Several PTMs have been shown to increase the stability and binding efficiency of *ICE* genes to downstream genes for instance: phosphorylation, ubiquitination, and SUMOylation [25]. Phosphorylation is one of the most vital post-translational modifications of *ICE* genes, regulating the cold stress tolerance through the actions of the *OPEN STOMATA 1* (*OST1*) and other various transcription factors. The *OST1* mediates the *ICEs* and *CBFs* in various ways. *OST1* has been demonstrated to phosphorylate the *ICE1* in Arabidopsis and rice for stability, by binding to the *HIGH EXPRESION OF OSMOTICALLY RESPONSIVE GENE 1* (*HOS1*) to prevent the degradation of *ICE1* by *HOS1*. Furthermore, the *OST1* regulates *CBF* gene expression by phosphorylating *BASIC TRANSCRIPTION FACTOR* 3 (*BTF3*), a binding substrate to *CBF* genes [24]. Moreover, kinases within the *MITOGEN-ACTIVATED PROTEIN KINASE* (*MAPK*) cascade play an essential role in the phosphorylation of *ICE* genes; *ICE1* is phosphorylated at the Ser403 for stability and *CBF* regulation activation in the *MAPK* cascade [26]. In potatoes, *SaMMK2*, a constitutive kinase, was recently shown to positively promote the expression of *SaCBF* under cold stress, leading to cold stress tolerance through expression activation of *COR* genes [27].

Ubiquitination-regulated turnover of the *ICE*-*CBF* proteins improves cold stress tolerances in plants [28]. Little has been shown recently on the ubiquitination mechanism regulating the *ICE-CBF-COR* cascade. However, a *PUTATIVE U-BOX* type E3 ligase gene in grapevine, *VpPUB25/26*, was shown to promote the accumulation of *VpICE*1 and suppress the expression of *VpHOS1* [29]. *PUB25/26* was demonstrated to degrade the *MYB15*, an inhibitor of the *ICE*-*CBF* pathway during cold stress, thereby increasing the expression of *ICE1* [30]. Additionally, *SINA*, a ubiquitin ligase in bananas was also reported to increase the stability of *MaICE1* and to improve transcriptional activation of the *CBF* regulon [31].

In addition to PTMS, the *ICE*-*CBF* is also regulated by the hormonal responses of jasmonates (JA), ethylene, brassinosteroids (BR) [32], gibberellin (GA) [33], auxin, and salicylic acid (SA). Numerous auxin-related genes have been thoroughly discussed which include auxin biosynthetic genes (*CYP79B3* and *CYP83B1*) and auxin carrier genes (*LAX1/2*), and their down-regulatory effect in *CBF* expression [34]. Interestingly, exogenous treatment of several hormones on plants during cold stress has also been demonstrated to relieve the cold stress in plants. For instance, in the GA-*CBF* crosstalk, exogenous application of GA has been shown to regulate the over-expression of *CBFs* in dwarf plants, while underlying mechanisms still require more research. Other phytohormones are discussed in detail below.

Accumulating evidence has shown that most of the cold stress tolerances are due to the targets of *CBFs*, the *COR genes*. In Arabidopsis, more than 200 *COR genes* are either activated or repressed by the actions of the *CBF1/2/3* [11]. A myriad of *COR genes* has been identified and demonstrated to increase cold stress tolerance directly or indirectly in plants. These include the plant *Dehydrins* (*Dhns*), *late-embryogenesis-abundant* (*LEA*) *proteins*, *low-temperature induced proteins* (*LTIs*) and their products: anti-freeze proteins [35] osmo-regulators [36], chaperones, functional proteins, and kinases [17,18].

This review paper sums up recent studies and findings on the *ICE-CBF-COR* cold signaling pathway, discussing how plants continue to evolve for cold stress acclimation. These insights will enrich the plant stress response knowledge base, providing vital information on how to ameliorate plant losses due to cold stress in the wake of global climate change.

## 2. Conserved Motifs and Their Functionality in *CBF*, *ICE*, and *COR* Genes

### 2.1. C-Repeat Binding Factor/Dehydration-Responsive Elements Binding 1 (CBF/DREB1)

**Figure 1:**
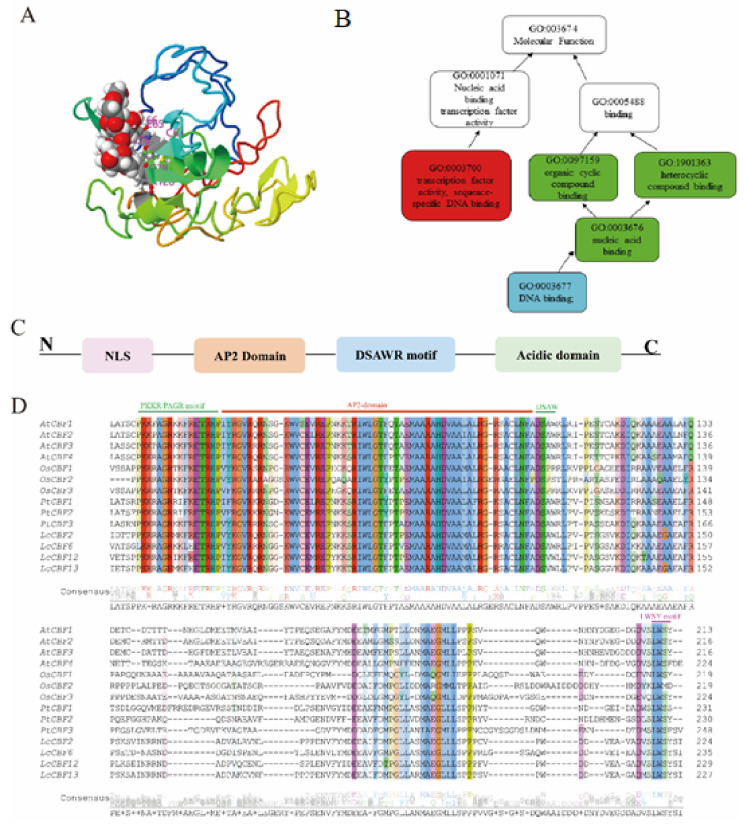
Structure, GO analysis, and sequence alignment of *CBF* in plants. (**A**) 3D prediction of *AtCBF1* secondary structure showing different domains denoted by different colors. (**B**) Gene ontology (GO) analysis of *CBF1* in Arabidopsis. (**C**) The schematic presentation of the AP2 structure, shows PKK/KPAGRxKFxETRHP, DSAWR, and LWSY motifs located upstream and downstream respectively, from the AP2 domain. These sequences contribute to the DNA binding specificity of *CBFs* to *COR genes*. (**D**) Multiple alignments of the amino acid sequences of *CBF/DREB1* proteins from different plant species. Different color schemes in the background show conserved amino acid sequences within the conserved AP2 DNA-binding domains, PKKR/PAGR, DASW, and LWSY, motifs. Characterized sequences include *AtCBF* (*Arabidopsis thaliana*), *OsCBF* (*Oryza sativa*), *LcCBF* (*Liriodendron chinense*), and *PtCBF* (*Populus trichocarpa*).

CBF transcription factors are involved in the cold signaling pathway in plants [24,37]. They were first discovered by Jofuku et al. [37] in *Arabidopsis* *thaliana* as plant-specific transcription factor types triggered by cold stress and/or the *ICE* [38]. *CBFs* belong to the superfamily of *APETALA2/Ethylene Responsive* (*AP2/ERF*) transcription factors, composed of *c.* 60 amino acid residues, and conferring a three-dimensional (3D) conformation arranged into a layer of three antiparallel β-sheets followed by a parallel α-helix sheet [39]. The 3D structure protein prediction analysis (Figure 1A) exposed Arg- and Try- residues within the β-sheet that link nucleotides of the binding site in the key groove of the DNA. Moreover, these key residues are well conserved in the *AP2/ERF* family [40]. The *Dehydration Responsive Binding Factor/C-repeat Binding Factor* (*DREB1/CBF)* family is distinguished by PKK/KPAGRxKFxETRHP, DSAWR sequence signatures, and an LSWY motif, schematically shown in Figure 1C. Medina et al. [41] first sequenced the *CBF* gene structure in Arabidopsis and revealed that *CBF1/2/3* genes are clustered on chromosome IV, with *CBF2* and *CBF3* located 3 and 7 kb downstream of *CBF1*, respectively [41]. In addition, they showed the presence of several regulatory sequences: the core CANNTG-consensus motif, the CACGTC-, and TACGTG-related sequences in their promoter regions [42]. CBFs (*CBF1/DREB1B, CBF2/DREB1C*, and CBF3/DREB1A) are known to bind to the *C-repeat/Dehydration Responsive Element* (*CRT/DRE*) sequence (TACCGCAT) in the promoters of *COR genes* for their transcription activation. Gene ontology (GO) analysis (Figure 1B) of *CBFs* revealed that their main molecular function is in the binding to cold-responsive genes for cold stress tolerance, through the *CRT/DRE* binding domains [43]. Recent reports have exhibited several *CBF* amino acid sequences from other plant species with a higher homology, carrying similarly conserved motifs (Figure 1D) [10,44,45]. Additionally, Novillo et al. [46] paraded a negative feedback mechanism of the *CBF/DREB1* transcription factors, that *CBF2/DREBIC* negatively regulates the expression of *CBF1/DREB1B* and *CBF3/DREB1A* in Arabidopsis. Likewise, overexpressed *CBF1/DREB1B* inhibits the accumulation of *CBF3/DREB1A* transcripts. However, mutational changes in *CBF2* (*cbf2*) enhance the collection of *CBF1/DREB1B* and *CBF3/DREB1B* transcripts leading to cold stress tolerance through the expression of *COR genes*. However, this negative feedback is essential for the accurate expression of cold regulatory genes in response to cold stress. 

Further Hannah et al. [47] demonstrated that there is an inherited relationship between the total number and expression levels of *CBFs* and cold stress tolerance [48,49] and that some *CBFs* are specific to dicots, while others are specific to monocots and exhibit different response patterns during cold stress [50]. Therefore, there is a shred of cumulative evidence on the functional roles of the CBFs in plants. Furthermore, *CBFs* have been expressed in several transgenic plants, and their effect revealed. Table 1 summarizes some of the *CBFs* that augmented cold stress in transgenic plants. Moreover, previous studies have demonstrated the functional roles of *CBF1/2/3* in Arabidopsis to bind to the promoters of target *COR genes* (*COR15A, COR47, COR78, KIN1,* and *LTI78*), inducing their expression for cold stress regulation. They all concurred that *CBFs* are induced by *MYC*-like *bHLH* and *AtICE*2, via the *AtCAMTA3* promoter [51,52]. A recent study in Longan (*Dimocarpus longan*) has identified three novel *CBF* genes, *DlCBF1/2/3*, that bind the CRT/DRE cis-elements, inducing the expression of *AtRD29A, AtCOR15A, AtCOR47*, and AtKIN1 consequentially in plant cold acclimation [53].

A few reports have stated the equal importance of *CBF1/2/3* in Arabidopsis for cold tolerance, while other researchers have proposed that only *AtCBF2/3* play significant roles in cold stress tolerance [56,57,58]. Salvo et al. [67] also revealed the importance of *CBF1* in cold induced (CI) citrus cultivars, participating in natural cold stress tolerance by triggering the expression of downstream COR genes. They concluded that *CBF1* is essential for cold tolerance in citrus fruits. A recent study in Asian pears (*Pyrus pyrifolia*) has shown the functional roles of *PpyCBF3* for cold tolerance. They showed that expressed *PpyCBF2/3* were linked to the expression of *PpyCOR* genes (*PpyCOR47, PpyCOR15, PpyRD29A,* and *PpyKIN*). They expressed *PpyCBF 2/3* genes in transgenic Arabidopsis and augmented cold tolerance through the lowering of ROS species, and antioxidant gene activities, suggesting that *PpyCBF2* and *PpyCBF3* were responsible for the expression of *COR* genes [54]. Nevertheless, it can be inferred that the importance of *CBF* proteins depends on the plant species and all *CB*Fs are vital and unique in function for cold tolerance.

### 2.2. Inducer of CBF Expression (ICE)

**Figure 2:**
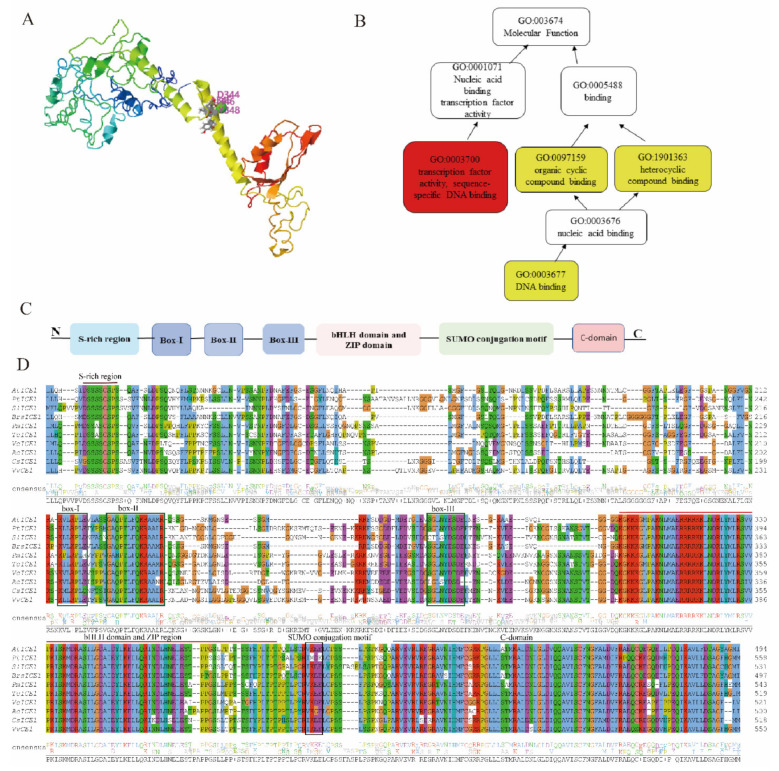
Structure, GO analysis, and sequence alignment of *ICE* genes in plants. (**A**) 3D prediction of *AtICE1* secondary structure, showing different domains denoted by different colors. (**B**) Gene ontology (GO) analysis of *ICE*1 in Arabidopsis. (**C**) A schematic presentation of *AtICE1*, depicting conserved binding domains and motifs. (**D**) Multiple alignments of *ICE* amino acid. Different color schemes in the background show conserved amino acid sequences within the conserved DNA-binding domains, the S-rich region, bHLH domain, the ZIP region, and the SUMO-conjugated motif in the *ICE1* proteins. Shown sequences have been characterized from *AtICE1* (*A. thaliana*), *SlICE1* (*S. lycopersicum*), *PtICE1* (*P. trifoliata*), *PmICE1* (*P. mume*), *VvICE1* (*V. vinera*), and *CsICE1* (*C. sinensis*).

The *ICE* is a forerunner in the cold acclimation process that acts upstream of the cold response cascade [68,69]. It belongs to the basic *Helix-Loop-Helix* (*bHLH*) family. The *bHLH* transcription factors regulate the expression of cold regulatory genes; they contain conserved *bHLH* binding domains at C-terminals, as shown in Figure 2C, for specific interactions with downstream cold regulatory genes. The *ICE* was reported to carry the *bHLH* binding domain, and its amino acid sequence in the basic region is highly similar to other *bHLH* proteins. The *ICE* proteins bind to the canonical *MYC* cis-elements (CANNTG) in the *CBF3/DREB1A* promoter, leading to the induction of *CBF/DREB1* regulon [70,71]. Two isoforms of the *ICE* protein have been identified in Arabidopsis, *ICE1*, and *ICE2*, consisting of 494 and 450 amino acids, respectively. Distinguished by the presence of an additional amino acid box in *ICE2*, towards the end of Box II (Figure 2C,D), modifying the conserved LPPT sequence, and also the absence of Box I in the *ICE1* genes [72]. The Glu- and Leu-rich regions of the *ICE2* localized in the exon part, form additional alpha-helices in the secondary structure (Figure 2A). Additionally, the structure of *ICE2* has more phosphorylation sites than *ICE1*, otherwise, their secondary structures are similar, and they both include four exons and three introns [73]. Gene ontology (GO) enrichment analysis of *AtICE1* (Figure 2B) showed that the *ICE1* binding sites are enriched in several categories including nucleic acid binding (GO:0001071), an organic cyclic compound binding site (GO: 0097159), and heterocyclic compound binding (GO: 1901363) [71]. Thus, its main molecular function is for binding downstream of *CBF* genes.

Badawi et al. [15] demonstrated that the *ICE1* is specific to monocots and *ICE*2 is specific to eudicots. However, other *ICE1*-like proteins are also present in dicots and they show high homology in the C-terminus region [69]. Many different types of *ICE*-like genes with homologous conserved domains have been recently revealed and expressed in various transgenic plants for tolerance investigation to cold stress (Table 2). Recently, Kashyap et al. [73] showed an *ICE* homolog, *BjICE53*, to be involved in the cold signaling pathway in *Brassica juncea*. They revealed conserved domains and motifs that bind to the CRT/DRE motifs of *BjCBF* for the expression of downstream *cold-regulatory genes* [74,75,76]. Another study in *Chrysanthemum morifolium*, “Jinba”, demonstrated that overexpression of *CmICE2* in transgenic Arabidopsis induces the expression of downstream cold regulatory genes (*AtCBF1/2, AtCOR6.6a/414, and AtKIN1*), leading to cold stress tolerance through increased proline contents, superoxide dismutase (SOD) activities, and elevating catalase (CAT) levels [77]. Zuo et al. [78] also revealed the biological roles of *ICE1* in *Zoysia japonica* (*ZjICE*1) to positively regulate the cold response signaling pathway. They disclosed that the overexpression of *ZjICE1* triggers the expression of cold regulatory genes (*ZjCBF1-3* and *ZjCOR47A*).

### 2.3. Cold Regulated (COR) Genes

**Figure 3:**
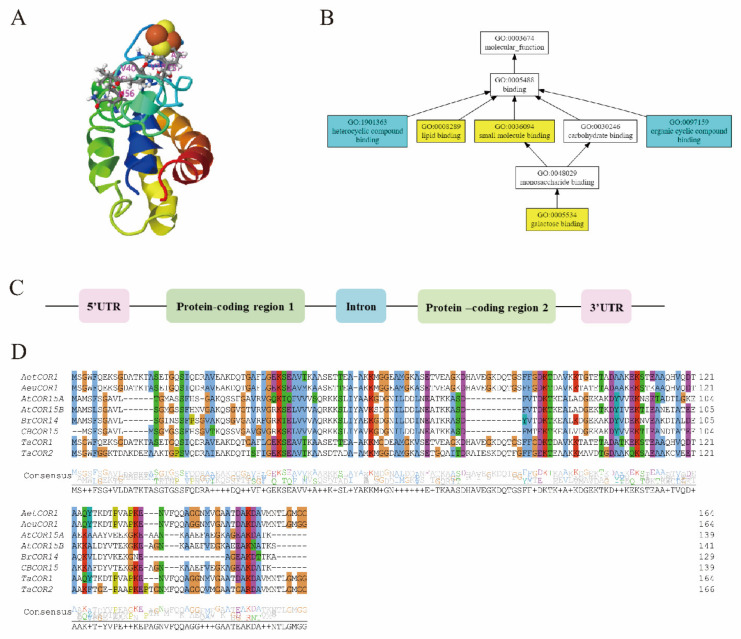
Structure, GO analysis, and sequence alignment of *COR* genes in plants. (**A**) 3D prediction of *AtCOR15A* secondary structure, showing different domains denoted by different colors and helices formed by different interactions of domains. (**B**) Gene ontology (GO) analysis of *AtCOR15A*. (**C**) The schematic presentation of plant *COR* genes with two flanking exons in the 5′UTR and the 3′UTR and a central intron. (**D**) Multiple amino acid sequence alignments of different *COR* genes. Different color schemes in the background show conserved amino acid sequences in different *COR* genes. Aligned sequences include: *AetCOR1, AeuCOR1, AtCRO15A/B, BrCOR15,* and *TaCOR1/2*.

Several reports have shown that cold-inducible genes designated as *Cold-responsive* or *Cold Regulated Genes* (*COR genes*), *ABA-inducible protein-coding* (*KIN1* and *KIN2*) [84], *Responsive to Desiccation* (*RD*), and *Low-Temperature-Induced (LTI) genes* carry the CRT/DRE cis-acting element augmenting cold stress tolerance through the *CBF*-dependent pathway [11]. *CBFs* bind to the *C-repeat* (*CRT/DRE*) cis-elements located in the promoters of *COR* genes denoted by a CCGAC sequence, further activating their transcription [85]. About 10–20% of the total *COR* genes in Arabidopsis are estimated to be directly regulated by *CBFs* [86]. Most studied *COR* gene structures are flanked by exons (protein-coding regions) localized both in the 5′UTR and 3′UTR with a central intron, schematically shown in Figure 3C. However, different *COR* gene families are distinguished by specific motifs, but all share a conserved *CRT/DRE* binding site that binds upstream of *CBF* genes for their expression [87]. Several *COR* genes in the cold signaling pathway have been characterized and some of their amino acid sequences are shown in Figure 3D, showing several conserved domains within the *COR* genes. Therefore, plants respond to cold stress in three discrete phases depending on the temperature range, that is, pre-hardening, hardening, and plant recovery [88]. Specific *COR* genes act to stabilize both membrane phospholipids, proteins, and cytoplasmic proteins, maintaining hydrophobic interactions, ion homeostasis, and scavenging ROS, depending on the temperature range [89,90].

Previously, different targets of the *CBF* genes were discussed fully. We will partially discuss a few *COR* gene families in this section. The *COR413* family has two distinct groups, *COR413*-*plasma membrane* (*COR413pm*), *COR413-inner membrane 1* (*COR413im1*), and *COR413-thylakoid membrane* (*COR413tm*) [91]. It is known that low temperature influences the structure of the plasma membrane by reducing the fluidity and increasing rigidity, with these changes leading to the expression of *COR413pm* genes. Recent studies have revealed that cold-induced *PsCOR413Pm2* [92] and *AtCOR413pm* [93] carry similarly conserved binding domains in their promoter regions. The *COR413pm* genes regulate cold stress through enhancing the Ca^2+^ influx and the expression of stress-related *COR* genes (*COR6.6, KIN2, COR15A, COR15B, COR47, and COR78/RD29*) and *CBF* (*CBF2* and *CBF3*) genes in Arabidopsis. These results suggest the interconnection with the cold-responsive genes, concurring with the *ICE-CBF-COR* cascade. While the *COR413im* localized in the inner-membrane was shown to activate the cold-expression of *COR15A* and *COR15B* in Arabidopsis, their expression mechanism still remains a mystery to be unrevealed [92].

*Dehydrins* (*DHNs*) are a subgroup of the *Late-Embryogenesis-Abundant* (*LEA*) proteins in angiosperms. They are characterized by high hydrophilicity and a diverse combination of typical domains. Most notably, the K-segment (EKKGIMDKIKEKLPG) sequence near the C- terminus. They accumulate in plants in response to cold stress, particularly, the SKn type, which protects the membrane from freeze desiccation by potential dehydration-induced demixing of membrane lipids, acting as molecular chaperones or ion sequestration [94]. For instance, the *AtCOR15A* with its secondary structure (Figure 3A), is suited for binding to other proteins and acts as a chaperone protecting the membrane from freeze desiccation. GO analysis of *COR15A* (Figure 3B) has provided supporting evidence on the molecular binding function of *AtCOR15A* to lipids, carbohydrates, heterocyclic compounds, and other small molecules [95]. Previous research evidenced *dehydrins* (*OsDhn1, lip5, and lip9*) to regulate cold stress through the *CBF* pathway in rice, and their homologs *Wcor410* and *AtCOR47*, which are both known to be regulated by *CBF1/DREB1B*. Apart from these aforementioned dehydrins, several *DHN* proteins have been shown to regulate cold stress through the *CBF* pathway including, *Wcs120, COR47*, and *RD17.* Recently, research has evidenced that *CBF1* identifies the consensus sequence (CCGAC) of the *CRT/DRE* elements from *Dehydrins* in *Vitis vinera* and Triticeae species [96].

*Low-Temperature Induced* proteins (*LTIs*) enable plants to acclimate during low but non-freezing temperatures. Two *LTIs* have been shown, *LTI78* and *LTI 65* in Arabidopsis, to regulate cold stress and carry a 9 bp conserved sequence (TACCGACAT) in their promoter regions, termed the dehydration-responsive element (DRE) (Figure 3D) [35]. *COR* genes have also been demonstrated to act as regulators of other cold regulatory genes. Recently, the *COR27/28* genes were reported to regulate the *COP1-HY5* regulatory hub influencing the freezing tolerance and the circadian clock. These genes interact directly with *HY5* promoters and regulate negatively the transcription of other *COR* genes promoting hypocotyl elongation in Arabidopsis [97]. Several *COR* genes have been expressed in different transgenic plants and their regulatory effect revealed. Table 3 below summarizes reports of different *COR* genes that were expressed in other transgenic plants.

## 3. *Mitogen-Activated Protein Kinase* (*MAPK*) Cascade and Hormonal Responses Regulating the *ICE-CBF-COR*

**Figure 4:**
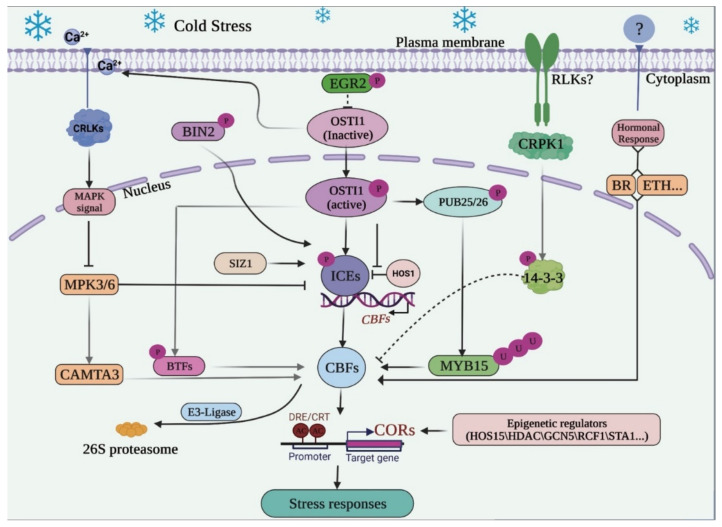
The *ICE-CBF-COR* response pathway to cold stress initiates at the plasma membrane to plant cold tolerance. Cold sensors localized in the plasma membrane sense cold stress and an influx of Ca^2+^ ions trigger the calcium downstream effector, *CRLKs* in the calcium response channel. Consecutively, triggering the *MAPK* cascade, through the activities of *MPK3/6,* and directly inhibiting the *ICE*2 and/or activating the *CBF* genes through the *CAMTA3*. Resulting in enhanced expression of *COR* genes and cold tolerance. Another receptor, *RLK* phosphorylates the *14-3-3*, stabilizing it for translocation into the nucleus, and inhibiting the *CBF* transcripts expression. Hormonal responses in the ethylene, BR, and JA hormones directly enhance the expression of *CBFs* through triggering various TFs. The *ICE* genes are further phosphorylated through several PTMs to regulate the expression of CBFs, sequentially regulating the expression of *COR* genes and cold stress response.

Putative sensors embedded in the plasma membrane such as the *OsCGNC14/16* in rice [102] and *AtNN1* in Arabidopsis [103] trigger Ca^2+^ influx in the cytosol and other cell organelles through Ca^2+^ channels as a secondary response to cold stress. Secondary messengers, Ca^2+^-dependent proteins, retort to cold stress, playing an imperative role in intracellular signal transduction [104]. They bind to several proteins (TFs, protein kinases, ion channels, and other enzymes) including calmodulins to execute their regulatory functions. Therefore, Ca^2+^/CaM-dependent proteins have been reported in various plants including: *Vitis vinifera* [105], *Zea mays* L [106], soybean [107], *Brassica napus* [108], *Populus trichocarpa* [109], citrus trees [110], and other Gossypium species [111]. Kinases and their profile expressions under cold stress have been recently reported in *Brassica napus* [112], *Jatropha curcas* [113], and *Common vetch* [114]. The *Mitogen-Activated Protein Kinase* (*MAPK*) cascade regulates cold stress through the binding roles of *Calcium/Calmodulin-Regulated Receptors Kinase-Like 1* (*CRKL1*) [115]. Research on the *MAPK* cascade in Arabidopsis has demonstrated that *CRLK1/2* interacts with the *MEKK1*, a *MAPK* module responding to lower temperatures [116]. Then, the *MEKK1* sequentially phosphorylates the *MKK2*, in turn activating *MPK4/6* [117], formulating a pathway upstream, *CRLK1-MEKK1-MKK2-MPK4-MPK3/6*, that enhances the expression of *CBF* genes [118]. Previous studies reported on an *MPK3/6-CBF* enhancing substrate, the *calmodium-binding transcriptional activator 3* (*CAMTA3*), a putative *MPK3/6* substrate with five phosphopeptides and *MAPK* phosphorylation sites, that activates *MAPKs* in the *MPK3/6-CAMTA3* module. The *CAMTA3* binds to the *CBF2* promoters to induce the expression of *COR* genes in Arabidopsis [119]. Put together, these entities model a series of phosphorylation reactions after the Ca^2+^ influx, the *Ca^2+/^CaM-CRLK1/2-MEKK1-MKK2-MKK2-MPK3/6-CAMTA3-CBF2*, to enhance the expression of *CBF2* and consequent downstream *COR* genes (Figure 4). Early research showed that the overexpression of *CAMTA3* induces the expression of *RD29A* and *COR6.6* through *CBF* regulations [120]. Likewise, a comprehensive set of experiments has shown the importance of this pathway in the induction of the *AtCBF2*, by proving that mutants of *mpk3, mpk5*, *and camta3* are freezing sensitive [121]. Meanwhile, another pathway, the *CRLK1/2-MKK4/5-MPK3/6*, negatively regulates cold stress tolerance by reducing *CBF* expression through inhibiting the *ICE* transcription (Figure 4). The *MPK3* binds to the promoters of *ICE1*, promoting its degradation and consequently reducing the transcription of the *CBFs* [122,123]. Nonetheless, both *mpk3* and *mpk6* mutants have been shown to increase *CBF* expression leading to cold stress resistance in plants [84]. Wholly, these two regulatory pathways may be viewed as a single negative feedback mechanism that regulates the expression of cold regulatory genes. In Arabidopsis, phosphorylated *MPK6* mediates the negative expression of *CBF3* by activating a negative regulator, *MYB15* [122]. On the contrary, *MPK4/6* activates *CBF* expression by inhibiting the *MKK4/5-MPK3/6* pathway [112] 

Likewise, the hormonal response controls vital biochemical regulatory processes in the *ICE-CBF-COR* cascade during plant cold stress. Important plant hormones in the cold signaling pathway are brassinosteroids (BR), jasmonates (JA), ethylene (Eth), and Abscisic acid (ABA) (which will not be discussed in this review). Jasmonate including its derivatives, methyl jasmonate (MeJA) and jasmonic acid are called jasmonates (JA). Cold stress in plants has been established to elevate endogenous jasmonates (JAs) biosynthesis. In the same manner, JAs also increase cold stress tolerance by interfering in the inhibitory effect of *JASMONATE ZIM-DOMAIN 1/2* (*JAZ 1/2*) proteins on the transcriptional activity of *CBFs* [124]. Recently, An et al. [125] demonstrated the role of *MdBBX37*, that is its binding effect to the *MdCBF* promoters activating the expression of *MdCBF* in the *BBX37-ICE*1-*CBF* module. Further analysis of this pathway in the rubber tree also exhibited that exogenous treatment with methyl jasmonate (MeJA) weaken the inhibition of *JAZ1/2* on the *HbICE2* transcriptional hub, resulting in the upregulation of *HbCBF1*, *HbCBF2,* and *HbCOR47*. These findings suggest that the relieved and expressed *HbICE2* prompt the expression of *HbCBF1* and consequently *HbCOR47*. In the rubber tree, *JAZ1/2* proteins bind to the *F-box* protein receptor (*COI1*), a ubiquitin ligase of the SCF complex, inhibiting the activation of *ICE2* and downstream genes, and in apple trees, *MdJAZ1/2* inhibit the binding of *MdBBX37* to *MdCBF1/4* reducing *CBF* expression [79,126]. However, cold-elevated endogenous jasmonic-acid levels in apple plants relieve the repressive effect of JA-repressors (*JAZ1-2*) on the *MdBBX37.* Exogenous application of MeJA and its regulating effect on cold stress has been demonstrated in several plant species including *C. annuum* [126], *Musca acuminate* [127], and Arabidopsis [124]. Previous studies showed the interaction of jasmonate with other hormones such as auxins and ethylene to regulate the *ICE-CBF-COR* cold signaling pathway [128,129]. For instance, in the JA-auxin crosstalk, IAA29 a type of auxin interferes with the *ICE-CBF-COR* pathway by inhibiting the inhibitory action of JAZs proteins on the *ICE2* and *CBF1* [124].

Like the jasmonates, cold alters the endogenous levels of ethylene, although the regulating effect of ethylene on the cold stress is inconsistent with various plant species. Ethylene has been demonstrated to alleviate cold stress in *G. max* [130], tomato [131], and grapevine [132], while in Arabidopsis [133] and *M. truncatula* [134], ethylene reduces cold stress tolerance [128]. Additional analyses in the ethylene response signaling pathway have suggested that ethylene regulates *CBF/DREB* expression through the action of *EIN3*, a transcription factor that binds the consensus sequence ATGYATNY [130,135]. In *G. max*, *EIN3* binds to the promoters of *CBFs* in the absence of ethylene reducing its transcriptional activity and expression of downstream *COR* genes [130]. However, exogenous treatment with an ethylene precursor (1-aminocyclopropane-1-carboxylate) and an ethylene biosynthesis inhibitor (amino-ethoxy vinyl glycine) were shown to increase and decrease cold tolerance, respectively. 1-aminocyclopropane-1-carboxylate augments the expression of *MdCBF1* through the mediating roles of ethylene response factors (*ERFs*) in the *MdERF1B-MdCIgHLH1-MdCBF1* pathway [135]. *ERFs* are known to bind *COR* genes (*CORLTRECOREATCOR15* and *MYBCORE*) cis-elements, enhancing freezing tolerance by reducing ROS species, and increasing SOD and POD levels [136]

As steroid hormones, brassinosteroids (BR) are synthesized from mass sterol campesterol through multiple hydroxylations and oxidations, further catalyzed with various cytochrome *P450* enzymes, including *DWARF4, CPD, ROT3*, and the *CYP85A2 BR6ox2* steroid, cumulatively known as BR-biosynthetic genes. BRs induce a multidirectional response in plants that include the regulation of cold-responsive genes (*ICE*s, *CBF*s, and *COR*s) and other hormonal cross-talks (ABA and JA) [137]. Nevertheless, cold treatment downregulates these BR-biosynthetic genes [32]. *BRASSINOSTEROID INSENSITIVE 2* (*BIN2*), a GSK3-like protein kinase form of brassinosteroids, a repressor and regulator in the BR-signaling is also known to target the *bHLH*-type proteins including the *ICE* genes. Ye et al. [138] recently showed that *BIN2* phosphorylates the *ICE1*, thereby reducing its stability and transcription of the CBF regulon. Further downstream the *BIN2* activities are controlled by acetylation roles of *histone deacetylase 6* (*HDA6* discussed below). The phosphorylated *ICE1* interacts with *HOS15* at the C-terminus further degrading *ICE*1 and attenuating CBF expression. Additional studies evidenced that *BIN2* activities are down-regulated in the early stages of cold stress by *HDA6* and later restored as a regulatory measure for *CBF* expression and levels [138]. Cold-induced BR also directly participates in the regulation of basal cold tolerance by increasing the expression levels of *CBF1/2/3*, *COR15A,* and *COR47-*like transcripts in *A. thaliana* [139]. Consistent with these findings, studies in tomatoes have suggested a BR component, brassinazole-resistant 1 (*BRZ1*), that inducts the expression of *CBFs*. They proposed that cold induces BR and *BRZ1* abundancies, then *BRZ1* binds to the E-box (CANNTG) and BRRE (CGTGT/CG) motifs in their promoters and increases the expression of downstream genes through the resultant *RBHO1* and hence cold stress tolerance. Further analysis demonstrated that *RBOH1* enhances *CBF* expression by altering the cold- and BR-induced accumulation in the redox-dependent system. The significance of the BR component, *BRZ1*, in the *ICE-CBF-COR* signaling pathway has been verified through the overexpression of mutant *brz1*, resulting in cold stress reduction and low expression levels of the *CBF* transcripts [140,141]. Furthermore, *CBF1* has been related to positive relief of chilling injury during post-harvest storage of tomato expressing *BRI1* and to decrease chilling injury tolerances in mutant BR synthesis CPD. Cold-induced BR biosynthetic gene in tomato, *SLCTP90B3*, has been established to bind the promoters of *CBF1* and induce its expression through the activation of the *ICE1* transcription hub [142]. 

## 4. Post-Transcriptional Regulations and Post-Translational Modification

Cold stress induces extensive post-transcriptional and post-translational-modifications (PTM) in several plants, affecting the quality and quantity of the mRNA and ultimately cold stress tolerance [143]. Thus, post-transcriptional regulations and PTMs regulate the expression of the entities in the *ICE-CBF-COR* signaling pathway. Two protein families regulate the developmental steps of post-transcription, the RNA binding proteins (RBPs) [144] and the RNA helicases [145]. The RBPs function as molecular chaperones, regulating alternative splicing (AS). AS events produce multiple transcripts from a single RNA and they transpire in specific mRNAs families of genes, affecting their normal gene transcription. Previously, AS was demonstrated to modulate *WDREB2* in wheat [146] and *MYB48/59* in Arabidopsis [147] affecting their binding efficiency to downstream *COR* genes. A recent study in tea (*Camellia sinensis*) has explored the impact of AS events on the *ICE-CBF-COR* genes. They reported that AS induces the expression of genes involved in the cold response signaling and their regulators including *CsbHLH1/2, CsMYBs*, and other *COR* genes, alleviating cold stress through the *CBF*-dependent pathways [148]. Although the mechanism by which *ICE-CBF-COR* genes are induced is still unclear. Chromatin remodeling changes the transcriptional activities of several *COR* genes during cold stress, rendering it more or less accessible to the transcriptional machinery [22]. Chromatin modification of *histone deacetylase 6/9* (*HDAC 6/9*) during cold stress links directly to the transcriptional activities and negatively regulates *COR* gene expressions [149,150,151]. Studies in rice have shown that *O. sativa HADCs* functional proteins positively regulate cold stress tolerance by activating *OsDREB1* expression, thereby enhancing cold stress through expression activation of downstream *COR* genes by the *CBF*s [152]. Epigenetic switches from a repressed state in chromatin models also regulate the expression of *COR* genes. *HOS15*, a *WD40*-repeat protein degrades *histone deacetylation 2C* (*HD2C*), modulating a complex (*HOS15-H2DC*) that deacetylases *COR* gene chromatin to repress gene expression. The *HOS15-H2DC* complex binds to the promoters of cold-responsive genes, for instance, *COR15* and *COR47* [153], and activates their expression, resulting in cold acclimation through the cold regulatory roles of these *COR* genes.

During post-translational modifications (PTMs), several genes and TFs interact with the *ICE*, *CBF,* and *COR* genes to modify their activity, conformation, localization, and stability. Phosphorylation, ubiquitination, and SUMO conjugations are major PTMs in plants regulating the cold stress response pathway [154]. Phosphorylation plays a significant role in plant cold acclimatization and is a reversible protein modification, with a high dependence on kinases and phosphatases. The most common phosphatase, *open stomata 1* (*OST1*) appertain for the SNF1-related protein kinase family and phosphorylates the entities in the *ICE*-*CBF* response pathway. The *OST1* interacts with E3-ubiquitin ligase (*HOS1*), thereby phosphorylating the *ICE1*, increasing its stability, and alleviating cold stress through inducing the activities of *CBF* genes [155]. Furthermore, variants of the mature polypeptide-associated complex of *OST1* phosphorylate the *BASIC TRANSCRIPTION FACTOR* 3 (*BTF3*) proteins, promoting their interaction with *CBF* proteins, and consequently increasing the stability of *CBF*s for efficient binding to *COR* genes downstream [123,156]. *OST1* has also been shown to interact with *PUB25/26* in the *OST1-PUB25/26-MYB15* pathway and to upregulate the expression of *CBF*s in Arabidopsis. The two U-box type ubiquitin ligases (*PUB25/26*) degrade *MYB15*, an inhibitor of *CBF*, thereby increasing the expression of *CBF* and *COR* genes [30]. A plasma membrane-localized receptor-like cytoplasmic kinase, *cold-responsive protein kinase 1* (*CRPK1*) phosphorylates the *14-3-3* genes, promoting their significance in the nucleus from the cytosol, coherently interacting with the *CBF* proteins, and reducing cold tolerance through destabilizing their binding affinity to *COR* genes [157].

Ubiquitination defines the rigorous action of three enzymes, E1 > E2 > E3. The E3-ubiquitin ligase plays the most vital role by interacting with the target molecule and providing scaffolding for the ubiquitination reaction. The number of ubiquitin molecules attached to a target molecule determines its fate, that is polyubiquitin, monoubiquitin, and ubiquitin [158]. Cold regulatory genes are affected by E3-ubiquitin ligases (polyubiquitination) that regulate their expression and cold stress tolerance. *HOS1*, a RING-finger E3 ubiquitin ligase participates in the negative feedback regulation of cold stress by mediating *ICE1* degradation at the onset of the cold stress response. However, mutant *hos1* expression enhances cold tolerance through loss-of-function [159]. CRISPR/cas9-mediated genomic loss of function studies have also revealed that the *hos1* provokes significant fluctuations in the expression of *ICE1* in Arabidopsis [160]. *HOS15*, a ubiquitin ligase interacts with *CBFs* and modulates their binding to the *COR* gene promoters through chromatin remodeling [161]. *ICE1* in *Eucalyptus camaldulnesis* interacts with *EcaHOS15* in the ubiquitination-proteasome pathway, increasing its binding affinity to *Ec*a*HOS1*. However, substitutional processes of serine (Ser158) by alanine (Ala) inhibit *EcaHOS15-EcaICE1* interaction leading to reduced binding efficiency of *CBF*s to *COR* genes. When bound to the *ICE*1, cold stress tolerance is enhanced through the enhanced expression of the *CBF*s [162].

SUMOylation a similar process to ubiquitination regulates cold stress through the action of SUMOs [163]. SUMOs are bound to a lysine residue of a target protein in three steps with three SUMO ligase enzymes (E1 > E2 > E3), provoking their interaction with target proteins and disturbing their PPIs with other proteins [154]. *SIZ1*, an E3 SUMO ligase has been demonstrated to positively increase freezing and cold stress tolerance in Arabidopsis by inhibiting *ICE1* ubiquitination. More specifically, SIZ1 sumolyates the *ICE1* at position K393, and additional results have proven that this sumoylation has no negative implications on the *ICE1* activity, but rather inhibits polyubiquitination of *ICE1* by the *HOS1*, decreasing *ICE*1 degradation and increasing *CBF3* expression. Moreover, the sumoylated *ICE1* negatively regulates the repressive actions of *MYB15* on *CBF3.* The loss-of-function of *SIZ1* has also been shown to reduce cold tolerance and increase *ICE*1 ubiquitination, concluding that *ICE*1 levels are determined by the balance of SUMOylation and ubiquitination processes [164,165].

## 5. Conclusions and Future Perspectives

The *ICE-CBF-COR* cascade plays a crucial role in the survival of plants during cold stress. Cold stress is perceived by plant sensors and other organelles: secondary responses induce the expressions of downstream *cold-responsive genes*. Various regulators, inducers, hormonal responses, post-transcriptional regulations, and/or the post-translational modifications induce the expressions of *ICE1/2*, and *CBF1/2/3* genes which in turn enhance the expression of *COR* genes. In detail, the *OST1, HOS15, MYB15*, the *MAPK* cascade and their direct and/or indirect regulation in the expressions of *ICE1* and *the CBF1/2/3*, cross-interlink and interact with the key players within the *ICE-CBF-COR* and regulate their expression and consequently cold acclimation. The sum of these mechanisms was discussed in this review, and collected insights concur in the sequential expression of *ICE*s, *CBFs,* and *COR* genes. Therefore, it can be concluded that the *ICE-CBF-COR* is the central pathway to which different transcription factors, regulators, proteins, physiological factors, and other manipulators interlink to enhance cold stress. Although expression of genes at different response levels may or may not follow the hierarchal steps in response, such as the CBF-independent pathway. 

Nonetheless, elaborate mechanisms and other additional regulators still require further analysis, to fully understand the effect of seasonal changes, hormonal imbalances, and gene transcriptional/translational on the expression of *ICE*, *CBF,* and *COR*s. This review summarized cold stress tolerances through the *CBF*-dependent pathway (Figure 4). Expression of the *CBFs* has been discussed fully, demonstrating the upstream enhancer *ICE* genes and their roles, and the roles of the *CBF* in inducing the expression of downstream *COR* genes. Nonetheless, cross-links and biochemical interactions within these sequential expressions are not facilely comprehended. Further studies on the *CBF*-dependent pathway are required to expose all the possible and included response factors. This will be important in gene engineering the cold response genes, to improve cold stress acclimation in cold stress-sensitive plants. However, there are few prospects in understanding the cold stress response in model plants such as Arabidopsis, rice, wheat, and other socio-economic plants. For instance, the identification of the *CHILLING-TOLERANCE DIVERGENCE 1* (*COLD1*) receptor and the *G-proteins* in Japonica rice (not discussed in this review paper). The knowledge of their interaction with the *ICE-CBF-COR* cascade has improved the understanding of cold stress signaling in plants. Different transgenic plants have been manipulated to improve the expression of the *ICE*, *CBF* genes, and ultimately cold stress tolerance with the introduction of the *COLD1* receptor and interaction improvements with *G-proteins*. Furthermore, techniques such as CRISPR have knocked out inhibitors and reducers, reducing the *ICE*s, *CBFs*, and *CORs* expression, leading to increased plant cold tolerance in plants and understanding of the importance of certain regulators and enhancers, suggesting the importance and urgency of further identification of other molecular factors and pathways that directly or indirectly interact with the *ICE-CBF-COR* pathway. There is still a need to further the understanding of hormonal responses and their effect on the *ICE*-*CBF-COR*, such as ethylene regulating *ICE-CBF-COR* in other plants, the *MAPK* cascade and its regulatory behavior in the cold signaling pathway, considering the antagonistic roles of the *MPK6/3* with *MPK4* in Arabidopsis. Other mechanisms such as the PTMs and post-transcriptional regulations require extensive research to fully understand the impact of alternative splicing, chromatin modifications, and methylation on the transcription and translation of *ICE*, *CBF*, and *COR* genes. 

Taking into consideration the impact of global climate change on the overall plant growth and yield, there is still an urgent need for intense research on the *ICE-CBF-COR* cascade to answer many questions that remain unanswered in the *ICE-CBF-COR* pathway and how it can be improved to ameliorate cold stress and improve plant yield and growth.

## Figures and Tables

**Table 1:** Transgenic plants developed by the overexpression of CBF genes.

Gene	Species	Transgenic Technique	Transgenic Plant	Effect	References
*DlCBF1-3*	*D. longan*	*Agrobacterium*-mediated transfer	*A. thaliana*	cold stress tolerance	[53]
*PpyCBF1-3*	*P. pyrifolia*	*Agrobacterium*-mediated transfer	*A. thaliana*	cold tolerance	[54]
*IbCBF3*	Sweet potato	*Agrobacterium*-mediated transfer	*S. tuberosum*	cold tolerance	[55]
*AtCBF3*	*A. thaliana*	*Agrobacterium*-mediated transfer	*S. melongena* L.	cold stress tolerance	[56]
*EgCBF3*	*E. guineensi*	*Agrobacterium*-mediated transfer	*L. esculenta*	freezing tolerance	[57]
*PpCBF3*	*P. pratensis* L.	*Agrobacterium*-mediated transfer	*A. thaliana*	freezing tolerance	[58]
*GmDREB1B*	*G. max*	*Agrobacterium*-mediated transfer	*G. max*	cold tolerance	[59]
*DaCBF7*	*D. antarctica*	*Agrobacterium*-mediated transfer	*O. sativa*	cold tolerance	[60]
*PpCBF1V*	*P. pratensis* L.	*Agrobacterium*-mediated transfer	*M. domestica*	cold tolerance	[61]
*AtCBF1*	*A. thaliana*	*Agrobacterium*-mediated transfer	*S. lycopersicum*	freezing tolerance cold tolerance	[62]
*OsDREB1B*	*O. sativa*	*Agrobacterium*-mediated transfer	*N. tabacum*	cold tolerance	[63]
*HvCBF4*	*H. vulgare*	*Agrobacterium*-mediated transfer	*O. sativa*	Regulates cold stress	[64]
*TaDREB2*	*T. aestivum*	*Agrobacterium*-mediated transfer	*Hordeum vulgare*	freezing tolerance	[65]
*BnCBF5/17*	*B. napus*	*Agrobacterium*-mediated transfer	*Brassica napus*	freezing tolerance	[66]

**Table 2:** The response of transgenic plants developed by overexpression of *ICE* homologous.

Gene	Species	Transgenic Technique	Transgenic Plant	Effect	References
*SiICE1/2*	*S. involucrata*	*Agrobacterium*-mediated transfer	Arabidopsis	cold tolerance	[75]
*AtICE1*	*A. thaliana*	*Agrobacterium*-mediated transfer	Indica rice	cold regulation	[21]
*CmICE2*	*C. morifolium*	*Agrobacterium*-mediated transfer	Arabidopsis	cold tolerance	[77]
*BjICE46/53*	*B. juncea*	*Agrobacterium*-mediated transfer	Arabidopsis	cold tolerance	[73]
*HbICE1/2*	*H. brasiliens*	*Agrobacterium*-mediated transfer	Arabidopsis	cold tolerance	[79]
*ZjICE2*	*Z. japonica*	*Agrobacterium*-mediated transfer	Arabidopsis	cold tolerance	[78]
*RsICE1*	*R. sativus*	*Agrobacterium*-mediated transfer	Rice	cold tolerance	[80]
*OsICE1/2*	*O. sativa*	*Agrobacterium*-mediated transfer	Arabidopsis	cold tolerance	[81]
*ZmmICE1*	*Z. mays*	*Agrobacterium*-mediated transfer	Arabidopsis	freezing tolerance	[82]
*SlICE1a*	*S. lycopersicum*	*Agrobacterium*-mediated transfer	Tobacco	cold tolerance	[83]
*TaICE41/87*	*T. aestivum*	*Agrobacterium*-mediated *tr*ansfer	Arabidopsis	freezing tolerance	[20]

**Table 3:** The transgenic plants developed by overexpression of COR genes.

Gene	Species	Transgenic Technique	Transgenic Plant	Effect	References
*LeCOR413PM2*	*L. esculanta*	*A. tumefaciens*	Tomato	cold tolerance	[97]
*AtCOR27/28*	*A. thaliana*	*A. tumefaciens*	Arabidopsis	freezing tolerance	[11]
*MfLEA3*	*M. falcata*	*A. tumefaciens*	Tobacco	cold tolerance	[98]
*SikCOR413PM1*	*S. involucrate*	*A. tumefaciens*	Tobacco	cold tolerance	[99]
*SiDHN*	*S. involucrata*	*A. tumefaciens*	Tomato	cold tolerance	[100]
*PsCOR413PM2*	*P. subulate*	*A. tumefaciens*	Arabidopsis	cold tolerance	[91]
*RcDhn5*	*R. catawbiense*	*A. tumefaciens*	Arabidopsis	freezing tolerance	[101]

## Data Availability

Not applicable.
